# The Value of Antiseptics in Surgery

**Published:** 1895-10

**Authors:** William Stanton

**Affiliations:** Varysburg, N. Y., Physician to the Wyoming County Hospital


### THE VALUE OF ANTISEPTICS IN SURGERY.[1]

By WILLIAM STANTON, M. D., Varysburg, N. Y.,
Physician to the Wyoming County Hospital.

JULY 10, 1894, Mr. L. was attempting to control a vicious stallion when the beast seized him by his left arm, taking it into his mouth far enough for the incisor teeth to pass beyond and hold the arm in the space between incisors and molars, puncturing it with the canines. Loosening once and tightening again, he inflicted eight punctures, four anteriorly, beginning above at about the space between the insertion of the deltoid and the external border of the biceps and extending downward, nearly following the outer edge of the biceps as it spreads out lower on the arm, the highest two anterior wounds joined, making a wound perhaps one and one-half inches long. The two others were about five-eighths of an inch long and did not differ materially from any punctured wound, and none were more than one-half or five-eighths of an inch deep, as will be shown further on. The posterior wounds, made by the lower canines, were smaller and located over the triceps, averaging about half an inch in length. Hemorrhage was slight.

From the manner of their infliction, their location and the small external openings, the wounds, in and of themselves, could not have been serious. I was absent at the time of the injury, so a non-graduate dressed the wound and continued in attendance until the 14th. His treatment I relate as described by the patient and his family:

A solution, said to contain carbolic acid, was used with a cloth, to wash the arm and wounds sufficiently to remove the fine-cut tobacco which had been applied; a cloth, wide enough to encompass the arm, was then drawn under and held together above by safety pins, so as to be easily opened, to allow the attendants to frequently pour in a little of an "oil carbolic," which he left. Hot steamed bran-bags were wrapped around the arm and renewed hourly for thirty-six hours. Each morning the dressings were opened and by squeezing a cloth wet in a solution, whose nature was not explained, it was allowed to run gently over the wounds and the arm, a little more "oil carbolic" was poured on and the cloth was replaced.

After a time, "proud flesh" began to appear in the largest wound, when a narrow strip of cloth, an ordinary carpet rag, was tied tightly around the arm and the knot was brought over the granulations "to keep them down." July 12th, the family became aware of a bad odor and by the 14th it had become unbearable, no one being able to remain

1. Read and patient exhibited before the Wyoming County Medical Association.

long with the patient at one time. The arm was turning black and producing an abundance of pus, and, in response to anxious inquiries, the attendant calmly explained that the black part would slough off in a few days, that there was always "such a smell to such things," that it was "beginning to run nicely" and that the arm and patient were both "doing as well as could be expected." The patient was taking, every three hours, a capsule containing "caffenol," or some similar powder, two of which remained weighed 18 and 19 grains respectively. The "oil carbolic" appeared like rancid olive oil, revealing no odor or taste of carbolic acid.

July 14th, 10 P. M., I was called to take charge of the case. The patient had been having slight rigors all day; pulse 112 and irregular; temperature 106° F.; tongue swollen and very foul; abdomen tympanitic and tender. His arm lay in a trough, formed by the cloth arranged as described above, and saturated with putrid pus, which was running out at both the top and bottom; his shirt was saturated with the same to nearly the median line, both in front and behind, and on his side had produced an excoriation considerably larger than a silver dollar. A sheet, folded in about sixteen layers, supported his arm. The pus had saturated this, the feather bed and had soaked a patch the size of a tea plate on the mattress. The patient was lying on his back, causing the pus to gravitate posteriorly.

With a 1-2000 solution of corrosive sublimate I washed off the pus and found, with its longest measurement transverse to the arm, a patch of gangrene, six and a half inches long, four inches wide externally, four and a half inches in the median line and three inches wide internally. Under this was a cavity containing fully four ounces of pus, which had burrowed in and between the muscles in all directions from the gangrenous tract. Below, the integument and superficial tissues were loosened nearly to the bend of the elbow. Above they were loosened comparatively little, probably about an inch. Externally, it had lifted the superficial tissues fully two inches beyond the external line of the gangrene. Internally, it had gone through the biceps deeply, leaving a cavity considerably larger than a butternut, uncovering the brachial artery, and posteriorly had worked inside the aponeurosis of the triceps, probably extending from the deep cavity in the biceps, following close to the humerus, thus gaining the upper surface of the fascia, which holding the pus, allowed it to form a cavity in the triceps, extending fully four inches lengthwise of the muscle and about two inches wide. Besides this wholesale destruction of tissue, there were several sinuses, leading in different directions, into which a probe would pass from one to three inches, and in two or three it passed entirely through, emerging at some other point. On the surface, bounding the gangrene on all sides, was an excoriated strip, varying in width from half an inch above to one and one-half and two inches below, while internally and externally it extended so as to nearly girdle the arm. The hand, forearm, arm and shoulder were greatly swollen, the nails were blue, the hand and forearm pale and quite cold, while above the gangrene were great heat and redness. The axillary glands were somewhat enlarged.

My treatment consisted in first evacuating the pus, irrigating as thoroughly as possible with a hot sublimated solution, 1-2000, using a fountain syringe for the purpose, and inserting rubber drainage-tubes where it seemed necessary, using four, which remained in place an average of four and three-fourths days, the extremes being two and seven. The following day I began cutting away the dead tissues and removed a large portion that day. At the site of the injury the slough only averaged about five-eighths inch in thickness, and after its removal there remained no trace of the original punctures, which proves they were not deep. On removing the slough internally the brachial artery, with its attendant vessels, could be plainly felt and seen near the bottom of the cavity, their sheath covered with shreds of the destroyed tissues and entirely dissected out. The cephalic vein and one or two branches were destroyed and removed with the sloughs, and several small nerves left uncovered were cut away. Poultices of flax seed and charcoal were freely applied. The line of demarkation formed rapidly, allowing quick and complete removal of the slough. The circulation of the forearm and hand improved and the tumefaction of neighboring parts rapidly disappeared. I was never able to find any communication between the cavity in the biceps and that in the triceps, but feel sure it must have existed, for while the drainage-tube in the biceps remained in place the cavity in the triceps gave no trouble ; in fact, I did not realise its proportions until a day or two after removing this tube, when a little increased heat and swelling indicated the need of drainage. I then opened freely, making an incision two inches long through the triceps to the humerus. I got but little pus, but removed several shreds of fascia two and one-half to three and one-half inches long by one-fourth to one-half inch wide. I dusted the wound with powdered charcoal until a clean surface appeared, then used occasionally a little boracic acid, but usually simply cleaned and dressed with rubber protective and absorbent cotton, or sublimated gauze, or both. In seven days we had a healthy granulating surface. The pockets under the superficial tissues soon closed up and the large cavity in the biceps filled very rapidly, granulations first appearing at the bottom, covering up the exposed brachial artery. The hot sublimated irrigations were continued as long as any pockets or sinuses could be found. In two weeks I began skin grafting, placing in all thirty-eight grafts, of which thirty-four lived, being about 89 per cent., and by excluding nine taken from the patient, we have alive twenty-seven of twenty-nine taken from two young men, about 93 per cent. In thirty-four days from my first visit the entire surface was healed, excepting the incision in the triceps, which had purposely been kept open.

Six hours after my first visit, when you remember his pulse was 112 and his temperature 106°, his pulse had dropped to 86 and his temperature to 101°, and for the next twenty-four hours his pulse ranged from 86 to 100, averaging 92; temperature 101° to 104 3-5°, averaging 102 2-5°. July 16th, the second twenty-four hours, average pulse, 94; temperature 101 4-5°; July 17th, average pulse, 91; temperature 101 1-5°; July 18th, average pulse, 87; temperature 100 4-5°; July 19th and 20th, average pulse, 86; temperature 100°.

From the 20th to the 25th witnessed a steady decline to normal. The tympanites and tenderness gradually disappeared. The tongue cleared off and by the end of the first ten days the patient was much improved and had a good appetite. He complained of very little pain, and my principal medication was quinine and the syrup of the iodide of iron. As antiseptics, I used corrosive sublimate for irrigating and gauze and listerine as deodorizers.

I have brought this case to your attention, believing it to be of value, furnishing, as it does, two such important lessons. First, it shows what serious conditions may follow a slight injury when not properly cared for; and, second, the satisfactory results which may be achieved, even in severe cases, by thorough and careful antiseptic treatment. It is, perhaps, more convincing than a very serious injury which has proper antiseptic treatment from the start. Let me recall to your minds, for example, the terrible injury which many of us witnessed at Warsaw, in which there was such extensive injury to the superficial tissues and cranial viscera, together with a penetrating wound of the anterior fossa through the frontal bone.[1] Recovery, you remember, was rapid under rigid antiseptics. Compare this with the case just reported. Eight little punctures, not averaging over five-eighths of an inch long, piercing no more important structures than the integument, only four days and eight hours without antiseptics and we have twenty-six square inches of surface gangrenous, the odor of putrefaction everywhere and a supply of putrid pus, which, like lava from a live volcano, carried destruction to everything in its way, the arm literally riddled and dissected, a man with septicemia, pyemia and death staring him in the face. But, even at this advanced stage, with such extensive loss of tissue, notice the immediate and wonderful effects of antiseptics. A fall in temperature of 5° in six hours. Actual measurement showed there was no further extension of

---

1. Dr. Lusk, in *International Journal of Surgery*, December, 1893.

gangrene and in seven days we had in its place a healthy, granulating surface, and in twenty-seven days more, by the aid of grafts, we found the entire twenty-six inches of surface healed.

I believe the original wounds should have healed by first intention and I am satisfied that with one thorough irrigation with hot sublimated water and, possibly, a few stitches followed by one or two intelligent aseptic dressings, they would have healed without loss of tissue or pus.

